# The influence of rainfall and tillage on wheat yield parameters and weed population in monoculture versus rotation systems

**DOI:** 10.1038/s41598-021-00934-y

**Published:** 2021-11-12

**Authors:** María Luisa Gandía, Juan Pablo Del Monte, José Luis Tenorio, María Inés Santín-Montanyá

**Affiliations:** 1 Department of Environment and Agronomy, INIA-CSIC, Madrid, Spain; 2 Department of Agrarian Production, ETSIAAB, UPM, Madrid, Spain

**Keywords:** Plant sciences, Climate sciences, Ecology

## Abstract

Extreme climate events (ECEs) of drought are becoming common in Mediterranean areas and farmers need adapt agricultural practices to achieve sustainability. This field study took place in to gain insight into the effects of seasonal rainfall, tillage and crop systems on wheat yield and weed parameters. Conventional (CT), minimum (MT) and no-tillage (NT) systems in wheat monoculture and rotation cropping systems were tested during 3 years of study (2014–2015, 2015–2016 and 2016–2017). Growing Season Rainfall (GSR) was the most influential factor on yield parameters and weed population. In 2016–2017, categorized as an extreme climate event by drought, the GSR accounted for 43.4% of the historical average. This year, the wheat yield (373 kg ha^−1^) and harvest index (0.18) were the lowest. In 2015–2016, scarcer autumn rainfall (44 mm) affected the weed germination period, reducing the density (17 plants m^−2^) and diversity of weed species (3 species m^−2^) while yield was favoured by high winter and spring rainfall (247 mm). Our study revealed that tillage effects was not significant on wheat yield, but NT systems consistently showed higher weed density and diversity than CT and MT despite the irregular GSR during this study. The rotation system presented higher values of wheat grain yield (781 kg/ha) and dry straw biomass (1803 kg/ha) but also weed biomass (48.54 g m^−2^) compared to monoculture (27.50 g m^−2^). NT and rotation combined increased the weed community although did not reduce the wheat yield compare to conventional systems even with an ECE of drought.

## Introduction

In any agro-ecosystem, both the germination of plants and their subsequent development will depend on environmental conditions (soil and climate) as well as on land management techniques. In Mediterranean regions, it is quite common that annual rainfall varies, and distribution is irregular from year to year^1^.

It is agreed that the frequency and magnitude of global extreme precipitation events have increased, and more severe and longer droughts at regional level occur during the twenty-first century^2,3^. In the Mediterranean region, the increasing frequency of drought had a stronger influence on the development of agroecosystems than average climatic conditions^4^, affecting the crop yields and the biodiversity of agro-ecosystems^5^. The likelihood of an ECE occurring is becoming more probable and the impact of such events has the ability to disrupt agricultural systems^6^.

In agricultural semi-arid areas of Spain, it has been reported that tillage system plays an important role in buffering the effects of wildly varying precipitation levels in rainfed cereal agroecosystems^7^. At present, practices of intensive tillage systems are changing to conservation agriculture techniques, based on minimal disturbance of soil associated with stubble retention and diversification of crop species^8^.

These techniques, such minimum tillage (MT) and no-tillage (NT), facing the soil erosion problem^9^, reducing energy use and C emissions^10,11^, enhancing wildlife habitat and soil biodiversity^12^, and saving labour and time^13^.

Much research has reported that the adoption of MT and/or NT often had positive effects on cereal yield^14–17^ and weed control^18,19^. In conservation agriculture, the stubble left on the soil increased its water retention, particularly during dry years, compared to conventional tillage systems^20,21^. Malhi and Lemke^22^ showed that crop yield increased with NT in part due to conserve soil moisture. Our research group also found less competition for water from weeds in NT systems^23^. In other rainfed areas, wheat yield was positively affected by available water content in MT systems^24^. Other studies have shown that conventional tillage systems (CT) and erratic rainfall affected both crop yields and weed community in semi-arid areas of Spain. López-Bellido et al.^25^ and Cantero-Martínez et al.^26^ observed an increase in cereal grain yields under CT in wetter conditions, but not in the driest conditions. An excess of rainfall and soil water availability favoured the weed community hindering wheat yield production under conservation techniques^27^.

In addition, the intensive conventional crop production trend has promoted growing cereals in short rotations, even in monoculture^28–31^. Nevertheless, monoculture cereal systems could reduce productivity in dry areas. Wheat-based monoculture is common in the Mediterranean region^32^ and the growth cycle of winter wheat, which is the most important rainfed crop in our area, is affected by drought. It is known that a well-planned crop rotation (with the adoption of legume and/or cruciferous crops within the cereal rotation scheme) can increase the sustainability of the system in dry regions of the Mediterranean basin^33–35^. From national to regional spatial scales, growing a greater diversity of crops increases the stability of the regional harvest of all crops combined, acting as a buffer to climate variability^36^. All these findings highlighted the site-specific significance linked to climatic conditions, soil characteristics, management practices, agronomic history, and duration of experiments^37^.

In view of the foregoing, research initiatives are necessary under local conditions^38^. We hypothesised that NT combined to rotation systems would maintain the yield resilience, as well as increase the biodiversity, in those agroecosystems affected by ECEs of drought. Although there is a lot of information about tillage and crop systems available, there is scarce information about how the ECEs by drought can affect weed and crop dynamics differently, in the short-term. The objective of this study was to compare wheat yield parameters and weed populations dynamics in two crop systems (monoculture vs. rotation) and three different soil management systems (CT, MT and NT) over 3 years with different rainfall patterns, one of them characterized as an ECE by drought.

## Material and methods

### Site description and climatic data

Information discussed in this paper was obtained over 3 years, 2014–2015, 2015–2016 and 2016–202,017 from a long-term study (initiated in 1994), at the experimental farm of INIA “La Canaleja” located in Alcalá de Henares (Madrid, Spain: 40° 32′ N and 3° 20′ W; 600 m). The soil is a sandy-loam Calcic Haploxeralf (Soil Survey, 2014). Initial soil organic carbon content was around 7 g kg^−1^, which indicates a low organic matter content (1.1%) with low fertility (total N 0.077%) and a pH of 8.

This long-term trial is characterized by Mediterranean semi-arid climate. The historical average rainfall recorded in our region (1957–2000) and our experimental farm from 1994 to 2013 during the growing season (from October to June) were coincident, 372.1 mm and 374.5 mm respectively (Table 1). The mean historical temperature showed a marked seasonality, with mean values of 1.07 °C in winter months and 22.8 °C in spring months. The Growing Seasonal Rainfall (GSR) measurements during the 3 years of the study were grouped in quarters (Oct–Nov–Dec) = QR1; (Jan–Feb–Mar) = QR2 and (Apr–May–Jun) = QR3, the historical average was grouped in the same way. The maximum and minimum quarterly average temperatures for these years are shown in Table 1 as well as the historical average for the same quarters.

**Table 1:** Seasonal and accumulate rainfall (mm), and max and min average temperatures during 3-years-study and historical values.

Year	Rainfall (mm)				Accumulated rainfall (mm)
	QR1	QR2	QR3	QR4	
2014–2015	145.3	70.2	50.0	24.6	265.5
2015–2016	44.1	100.8	146.7	23.1	291.6
2016–2017	108.3	43.1	52.0	64.1	203.4
(1994–2017)	133.2	94.8	112.5	34.0	374.5
(1957–2000)	139.2	107.4	125.5	50.97	372.1
	**T max (°C)**				
2014–2015	17.5	13.7	26.7	32.4	
2015–2016	18.3	13.6	23.6	33.4	
2016–2017	17.0	14.6	28.1	32.2	
(1957–2000)	14.9	12.7	22.8	30.4	
	**T min (°C)**				
2014–2015	4.7	0.5	10.2	16.0	
2015–2016	4.9	2.1	8.8	15.6	
2016–2017	5.0	1.8	10.6	15.1	
(1957–2000)	3.7	1.07	7.97	13.2	

QR1 = (Oct–Nov–Dec); QR2 = (Jan–Feb–Mar); QR3 = (Apr–May–Jun); QR4 = (Jul–Aug–Sep).

*QR* quarterly, *T* Temperature.

### Tillage treatments and crop management

Three types of tillage system were used: no-tillage (NT); chisel ploughing (minimum tillage at 15 cm depth, MT) and mouldboard ploughing (conventional tillage at 30 cm depth, CT). Two crop systems were compared: monoculture of wheat and winter cereals rotated with legume and fallow. The rotation scheme consisted of fallow, wheat (*Triticum aestivum* L. ‘Marius’), legume (*Vicia sativa* L. ‘Senda’) and barley (*Hordeum vulgare* L. ‘Vinagrosa’. The analysis was performed on wheat crop plots, the common crop in both crop systems.

In pre-sowing, the residue management for each soil tillage system was the standard used in this area: mouldboard ploughing in CT, chisel ploughing in MT, and herbicide treatment with glyphosate (3 l ha^−1^) in NT plots were applied 15–20 day before sowing. The wheat sowing rate, 210 kg ha^−1^, was chosen according to standard seed density in the geographical area. After wheat harvesting, all crop residues were left on the surface, independent of the tillage system.

An integrated fertilizer application was calculated for each cereal plot, and applied yearly at sowing time, in which we analysed total N content in soil determined by Kjeldahl method (soil samples in each plot were taken at 10–20 cm deep). We calculated the N to be applied per plot by taking the sampled N content of soil (%) and N necessary for wheat crop (26 kg N 1000 kg^−1^) for an expected wheat grain yield of 3000 kg ha^−1^. We estimated the mineralization rate in our conditions to be 10%.

### Experimental design and data collection

The experiment, which began in 1994, consisted of 60 trials (split-plot design), divided into three tillage systems where cereal crops were tested in several rotation schemes with 4 blocks^39^. During the years of study, the experimental design consisted of two factors (tillage and crop systems) divided in a randomized split-plot design with 4 blocks. The four blocks were divided into three main plots (three tillage treatments fixed and repeated on the same plot during the experiment period) allocated randomly. Each main plot was split in two crop systems (monoculture of wheat and winter cereals rotated with fallow and vetch). The 24 subplots were coordinated to ensure that there was always a subplot of each type available for study.. Each plot was 12.5 m wide and 25 m long, and to prevent the edge effect, blocks were separated by 10 m between them.

Every year, wheat grain yield was determined at harvest time (June) with a micro-harvester, and grain yield was standardized to 12.5% moisture content. Additionally, before harvest, in all plots, three samples of wheat plants were manually collected (0.7 × 0.7 m) from a randomized distribution to determine the grain yield and the straw biomass. The resulting straw biomass was dried in an oven at 65°–70° for 48 h. Finally, the harvest index (HI) was calculated for wheat crop^40^.

Formula 1 $$ {\text{Harvest}}\;{\text{Index}}:\;{\text{HI}} = {\text{GW}}/\left( {{\text{GW}} + {\text{SW}}} \right) $$

where GW is grain weight and SW is straw weight.

Weeds were identified and counted by species in four random samples per subplot (0.25 × 0.25 m) every year. Sampling took place at the end of March every year, corresponding to general tillering of wheat (stage 22–25 BBCH) which is a crucial competition stage for weeds^41^. Weeds were oven-dried at 80 °C for 48 h, and dry weed biomass in each plot was determined.

Weed diversity indices (richness and evenness) were calculated. Shannon index (Formula 1) as the number of species present, and evenness with Pielou index (Formula 2) that reveals whether the community is dominated by one particular species or whether all species are represented by approximately equal numbers^42^.

Formula 2 $$ {\text{Shannon's}}\;{\text{index}}\;{\text{for}}\;{\text{richness:}}\;{\text{H}} = \sum {{\text{pi}}\;\ln \;{\text{pi}}} $$

Formula 3 $$ {\text{Pielou's}}\;{\text{index}}\;{\text{for}}\;{\text{evenness:}}\;{\text{E}} = \left( { -^{.} {\text{pi}}\;\ln \;{\text{pi}}} \right)/\ln \;{\text{S}} $$

where pi is the proportion of individuals found in the ith species and S the number of species.

### Statistical procedures

The influence of tillage and crop system on yield parameters (grain wheat yield; straw biomass and harvest index, HI) and over weed communities (weed density, weed dry biomass and weed diversity) over the 3 years were analysed. Analysis of variance for wheat yield parameters and weed communities was carried out using generalized linear model (GLM), with years, tillage systems and crop systems as fixed factors. Means were separated using HSD Tukey’s test with 95% probability level (*p* < 0.05). All weed data were *square root* transformed prior to analysis to normalize the residuals.

Regarding the biodiversity studies, we carried out three different stages: (1) Shannon and Pielou’s indexes, which were calculated for all weed data using HSD Tukey’s test with 95% probability; and (2) the nine most prevalent weed species were assessed statistically with ANOVA (α = 0.05), and homogenous groups were identified with HSD Tukey’s test. (3) A canonical correspondence analysis (CCA) was carried out to evaluate the relationships between the weed community (formed by 9 selected weed species) and the factors year, tillage and crop systems. The first axis of our CCA is a linear combination of environmental factors that can best explain variation in species abundance^43^. Additional Correspondence Analysis (COA) was carried out to correlate the relationship between the sampling sites and environmental factors. Relationships were described based on soil management history, rainfall patterns or crops rotations in order to define tendencies. The R-Project software (*Vegan* and *Ade4*) packages were used for data processing.

The study complied with local and national guidelines.

## Results

Mediterranean rainfall is characterised by a sharp contrast between the total annual values recorded along the years, with prolonged dry periods and other intense rainy periods. The Growing Seasonal Rainfall (GSR), from October to June, varied markedly over the three years of the study, as well as the quarterly distribution (QR1, QR2, QR3), which is normal according to the historical mean data (see Table 1). In all years of the experiment, GSR was lower than the historical mean (371.2 mm). In the first two years, 2014–2015 and 2015–2016, the volumes collected were 28% and 22% respectively lower than the historical average. The third year (2016–2017) was an exceptionally dry year with a total rainfall of 45.3% less than the historical average, there was also more than a 20% variability between the season rainfall this year, therefore, it was categorised as ECE for drought.

The temporal rainfall pattern distribution throughout the crop cycle was also completely different for the three years. In the first year, 2014–2015, the highest rainfall occurred during the autumn months (QR1) and accounted for 54.7% of the total GSR. This was within 4% of the historical mean. On the contrary, winter and spring rainfall was lower, and particularly the spring season turned out to be exceptionally dry, only 37.8% of average GSR. During the 2015–2016 crop period, although the total rainfall was similar to the previous year the distribution was totally different. The autumn (QR1) was particularly dry, with just 15% of the total GSR, whereas spring (QR3) rainfall was high, 50.3% of the GSR and 17% higher than the historical average. The winter rainfall (QR2) of the second year of study was similar to the historical average. The third year (2016–2017) showed a rainfall distribution pattern similar to the first year (2014–2015), with 53.2% of rainfall during the autumn months (QR1) and a dry spring (QR3) at 25% of the total GSR. Again, winter rainfall (QR2) was lower than the first year of study and than the historical average.

### Influence of year, tillage and crop system on wheat yield parameters

Table 2 shows the effects of tillage and crop system on the wheat yield parameters grain yield and straw biomass. In general, the average wheat yield in our region did not exceed 2000 kg ha^−1^ (https://www.mapa.gob.es/es/), and the mean obtained in the INIA experimental farm was 1577 kg ha^−1^ ± 62.54 from 1994 to 2013. At the experiment, every year of the study has shown very low grain yield values due to the low rainfall. The year had a significant effect on average yield values; the first two years had low grain yield production of kg ha^−1^ (less than half of the expected rate) and the last year grain yield was exceptionally low. Significant differences in yield parameters were not observed between soil tillage practices during this short-term experiment. However, the crop systems (monoculture *vs.* rotation) had a significant impact on yields, with better results in the plots with rotation system.

**Table 2:** Analysis of variance results for years, tillage system, and crop system.

	Yield (kg/ha)	Straw biomass (kg/ha)	Harvest index (HI)
**Year**	***	n. s	***
2014–2015	856 a	1521	0.36 a
2015–2016	820 a	1451	0.36 a
2016–2017	373 b	1685	0.18 b
**Tillage**	n. s	n. s	**
CT	674	1731	0.28 b
MT	696	1390	0.33 a
NT	679	1537	0.30 ab
**System**	***	***	n. s
ROT	781 a	1803 a	0.30
MON	585 b	1303 b	0,31
Y*T	n. s	n. s	n. s
Y*S	n. s	n. s	n. s
T*S	n. s	n. s	n. s
Y*T*S	n. s	n. s	n. s

Mean values for yield, straw biomass and Harvest Index (HI).

Different letters in each column indicate a difference between treatments according to HSD Tukey’s Test with 95% probability level (*p* < 0.05); *** = 0.05; ** = 0.1; * = 0.5

*CT* conventional tillage, *MT* minimum tillage, *NT* no tillage, *ROT* rotation, *MON* monoculture, *Y* year, *T* tillage, *S* system.

Similar to wheat grain yield, only the crop system had a significant influence on straw biomass, producing a greater amount of biomass per unit area in those plots subjected to rotation. In 2016–2017, despite a low total GSR (203.4 mm), QR1 rainfall was high (108.3 mm—77.8% of the historical average and 53% of the GSR) and we found the straw biomass with no significant difference from the two first years of the study. However, we observed the lowest grain yield due to a dry winter and spring (QR2: 43.1 mm and 40.13% of the historical average and 21.1% GSR, QR3: 52 mm and 41.43% difference from average and 25.5% GSR). The grain yield was low this year despite high straw biomass because the autumn rainfall volume was similar to average—allowing for vegetative development, but the winter and spring rainfall was very low (ECE conditions of drought).

The relationship between yield and biomass production per unit area (Harvest Index) of each system is also shown in Table 2 and gives information about the efficiency of each system. In our study, it was observed that the years 2014–2015 and 2015–2016 turned out to be more efficient, both years had similar accumulated rainfall during the crop cycle although with a different distribution pattern. The year 2016–2017 was exceptionally dry and had the lowest harvest index value. Our results showed that HI was affected by tillage systems. Minimum tillage was the most efficient system according to the HI and conventional tillage was the least efficient system. This positive effect recorded in MT was detected in the short-term, despite of no significant differences was observed in yield and straw biomass between tillage systems. NT did not differ significantly from other two tillage systems. Regarding the crop systems, no significant differences were detected, and the interaction between factors had no significant differences on the HI.

### Influence of year, tillage and crop systems on weed parameters

Weed density was significantly affected by the year (Table 3). The year 2015–2016 showed significantly less weed density than the other two years. This year with a low autumn rainfall (less than the historical average) coincided with low weed germination during this period. We found that weed density was significantly affected by the tillage systems, noting that NT was the system with the highest density of weeds and it was significantly different from the other two tillage systems (CT and MT). Likewise, there were significant differences in the interaction between the year and tillage system, the last year (2016–2017) with NT showed the highest weed density. With respect to the crop system, we did not observe significant differences in weed density. However, in 2016–2017, we observed higher weed density in rotation than in monoculture, possibly as consequence of the high autumn rainfall (QR1) which allowed for extensive weed germination.

**Table 3:** Analysis of variance results for years, tillage system, and crop system.

	Weed density (nº pl/m^2^)	Weed biomass (g/m^2^)	Weed diversity (nº sp/m^2^)
**Year**	***	***	***
2014–2015	66 a	18.04 b	6 a
2015–2016	17 b	28.98 b	3 b
2016–2017	76 a	66.04 a	6 a
**Tillage**	***	***	**
CT	32 b	21.23 b	4 b
MT	42 b	22.48 b	4 b
NT	86 a	70.4 a	6 a
**System**	n. s	**	n. s
ROT	53	48.54 a	5
MON	52	27.50 b	4
Y × T	***	**	n. s
Y × S	*	**	n. s
T × S	n. s	n. s	n. s
Y × T × S	n. s	n. s	n. s

Mean values for weed density, weed biomass, and weed diversity.

Different letters in each column indicate a difference between treatments according to HSD Tukey’s Test with 95% probability level (*p* < 0.05); *** = 0.05; ** = 0.1; * = 0.5

*CT* conventional tillage, *MT* minimum tillage, *NT* no tillage, *ROT* rotation, *MON* monoculture, *Y* year, *T* tillage, *S* system.

The weed dry biomass (g m^−2^) was significantly influenced by GSR, by the tillage system and by the crop system; as well as by the interactions year × tillage and year × crop system. The highest biomass production of weeds was observed in 2016–2017, because of the high autumn rainfall (QR1) before mentioned, that favoured the increase of weed biomass. Rainfall during QR2 and QR3 was very low and as the crop was damaged by drought, the weed biomass maintained their levels of infestation.

Significant differences in weed biomass were recorded between tillage systems. NT reached the highest weed biomass compared to the other two systems (CT and MT). In 2015–2016 the lowest autumn rainfall (QR1) in NT plots led to the highest biomass of weed species compared to CT and MT plots. In the same way, when wheat was rotated, weed biomass was significantly higher than wheat monoculture, and significant differences between year and crop system interactions were observed. The conditions created by higher autumn rainfall in 2016–2017 could favoured some perennial weeds growth.

Weed diversity (Shannon and Pielou indices) was significantly influenced by the year and the tillage system (Table 4). There were significant differences between the year 2015–2016 and the other two years; this year showed the least diversity, probably due to the lack of rainfall during the autumn (QR1). We found that weed diversity in the NT system was significantly higher than CT and MT, with no significant differences between the latter two. No differences were detected based on crop systems, nor in any of the possible interactions. Pielou’s index only was significantly affected by tillage, we observed that CT produced more even population of weed species than MT and NT.

**Table 4:** Analysis of variance results for year, tillage system, and crop system.

	Shannon	Pielou
**Year**	**	n. s
2014–2015	1.103 a	0.726
2015–2016	0.820 b	0.813
2016–2017	1.080 a	0.733
**Tillage**	*	*
CT	1.019 ab	0.816 a
MT	0.850 b	0.720 b
NT	1.135 a	0.743 ab
**System**	n. s	n. s
ROT	1.006	0.75
MON	1.006	0.76
Y × T	n. s	n. s
Y × S	n. s	n. s
T × S	n. s	n. s
Y × T × S	n. s	n. s

Mean values for Shannon and Pielou index.

Different letters in each column indicate a difference between treatments according to Tukey’s HSD Test with 95% probability level (*p* < 0.05); *** = 0.05; ** = 0.1; * = 0.5

*CT* conventional tillage, *MT* minimum tillage, *NT* no tillage, *ROT* rotation, *MON* monoculture, *Y* year, *T* tillage, *S* system.

### Canonical correspondence analysis (CCA) and correspondence analysis (COA) analysis for main selected weed species

Nine predominant weed species were selected and the influence of environmental and management factors on their abundance were analysed (Table 5). The climatic conditions of the year had a significant effect on the presence and abundance of 7 of the 9 species. This influence was due to total rainfall and its distribution, clearly represented by the years 2015–2016 and 2016–2017, which were totally different in terms of volume and distribution rainfall. *Capsella bursa-pastoris* L., *Hypecoum spp*, *Papaver spp*. and *Lolium spp*. significantly decreased the year 2015–2016, despite of the most accumulated annual rainfall, the QR1 rainfall was the lowest. The contrary occurred to the increased *Cardaria draba* (L.) Desv. density.

**Table 5:** Analysis of variance results for years, tillage system, and crop system for weed density.

	*Anacyclus clavatus*	*Capsella bursa-pastoris*	*Cardaria draba*	*Fumaria spp.*	*Galium spp.*	*Hypecoum spp.*	*Papaver spp.*	*Lamium amplexicaule*	*Lolium spp.*
**Year**		*	***	**		**	***	**	*
2014–2015		0.750 ab	0.166 b	2.250 a		18.917 a	4.458 b	0.708 a	1.708 a
2015–2016	n. s	0.042 b	2.125 a	1.375 b	n. s	3.875 b	0.375 c	0.083 b	0.292 b
2016–2017		1.083 a	0.750 b	1.125 b		18.500 a	13.542 a	0.208 b	0.750 ab
**Tillage**	***	*	***		***	**	***	***	
CT	0.541 b	0.500 ab	2.250 a		0 b	6.417 b	4.625 b	0 b	
MT	0.208 b	0.166 b	0.583 b	n.s	0 b	13.125 ab	3.583 b	0 b	n. s
NT	1.541 a	0.208 a	0.208 b		2.75 a	21.750 a	10.167 a	1.01 a	
**System**	**		***	*					
MON	1.005 a	n. s	0.222 b	1.917 a	n. s	n. s	n. s	n. s	n. s
ROT	0.472 b		1.806 a	1.250 b					
Y × T	n. s	n. s	***	n. s	n. s	n. s	n. s	***	n. s
Y × S	n. s	n. s	***	n. s	n. s	n. s	n. s	n. s	n. s
S × T	***	n. s	***	n. s	n. s	n. s	n. s	n. s	n. s
Y × T × S	n. s	n. s	***	n. s	n. s	n. s	n. s	n. s	n. s

Mean values for nine main weed species.

Different letters in each column indicate a difference between treatments according to HSD Tukey’s Test with 95% probability level (*p* < 0.05); *** = 0.05; ** = 0.1; * = 0.5

*CT* conventional tillage, *MT* minimum tillage, *NT* no tillage, *ROT* rotation, *MON* monoculture, *Y* year, *T* tillage, *S* system.

The soil NT increased the density of six weed species (*Anacyclus clavatus* (Desf.), *Capsella bursa-pastoris* L, *Galium* spp, Hypecoum spp, *Lamium amplexicaule* L, and *Papaver* spp) which germinated on the superficial soil layer. *Cardaria*, with pivoting roots, increased in conventional tillage, and *Fumaria spp*. and *Lolium spp.* did not present significant differences between the different soil tillage techniques.

With respect to the crop system, only 3 species (*Anacyclus*, *Fumaria* and *Cardaria*) showed a significant relationship with the cropping system. *Anacyclus* and *Fumaria* were more abundant in monoculture than rotation systems, because the fallow before wheat facilitated those winter annual species control. The contrary occurred to *Cardaria*, which was better controlled by monoculture than rotation. The continuous wheat cropping competed with the pivoting roots of *Cardaria* by the deep water and space. We have also found a significant interaction between the tillage system and the cropping system in *Anacyclus*, with higher presence in NT and monoculture, and *Cardaria*, more abundant under CT and rotation systems. Significant interactions between the year and the tillage system were observed for *Cardaria* and *Lamium* being favoured by CT and NT systems respectively and, from a climatic point of view, these species were influenced by two different years. The rainiest year (2015–2016) facilitated *Cardaria* emergence and the high autumn rainfall in 2014–2015 favoured *Lamium* emergence.

**Figure 1:**
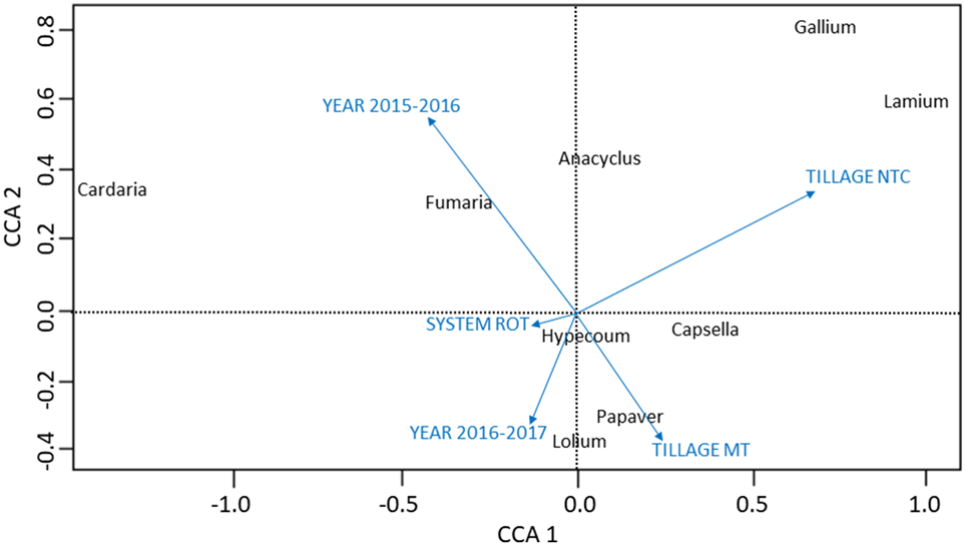
Ordination diagram of the reduce CCA model. *In black* = Weed species (only genus designation); *in blue* = the 5 significant explanatory variables. Only the species with the highest fit on the first two CCA axes are presented.

**Figure 2:**
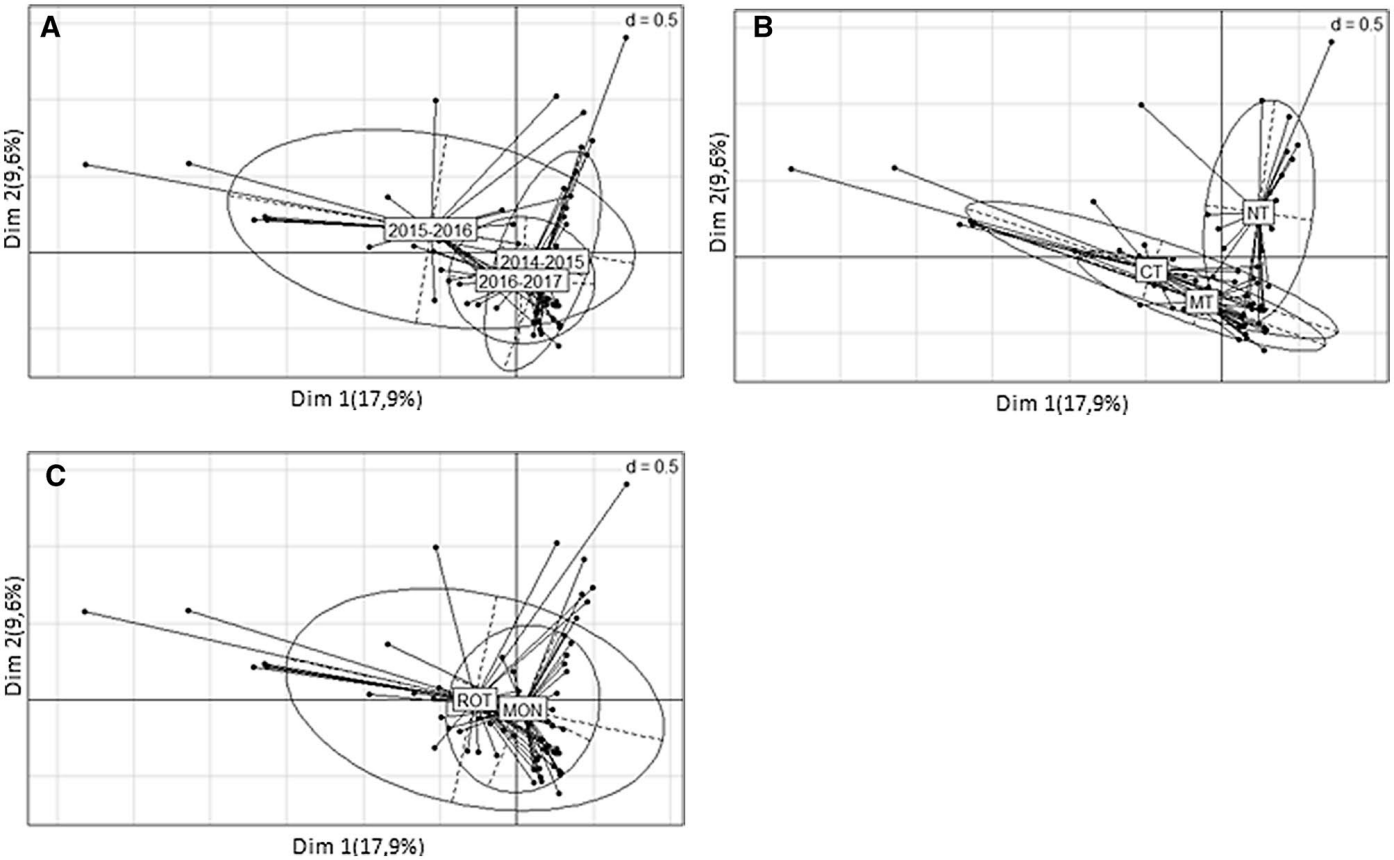
Correspondence analysis (COA) giving the coa1 duality diagrams (**A**, **B**, **C**). (**A**) = duality diagram with factor year; (**B**) = duality diagram with factor tillage; (**C**) = duality diagram with factor system. The values of d give the grid size.

Canonical correspondence analysis (CCA) and Correspondence analysis (COA) were carried out for the 9 main species and were reported in two ordiplots (Figs. 1 and 2). In the first ordiplot (Fig. 1), the main axes of CCA corresponded to 17.9% and 9.6% of the total variability. Although the cumulative variation between the first two dimensions did not explain a large amount of variation, in studies where a population was influenced by several factors, the randomness may be greater than the influence of the factors themselves^44^. This result showed that no single environmental factor alone influenced the dynamics of weed population in the circumstances in which our study was conducted.

As can be seen in Fig. 1, all nine weed species were highly affected by 5 discriminant elements: (1) no-tillage (0.751); (2) year 2015–2016 (− 0.623); (3) year 2016–2017 (0.120); (4) MT (− 0.253) and (5) rotation (ROT) system (− 0.283). No-tillage was the most discriminant element, positively affecting weed species: *Lamium* (0.998); *Galium* (0.815); *Capsella* (0.3712); *Papaver* (0.136) and to a lesser extent *Anacyclus* (0.08). *Cardaria* (− 1.35) and *Fumaria* (− 0.293) were negatively affected by year 2015–2016. *Lolium* was highly correlated with the MT. *Capsella* , *Papaver* and *Hypecoum.* were negatively affected by the year 2016–2017.

Figure 2 shows the Correspondence analysis (COA) expressed as ordiplots (A. B. C.). Each ordiplot, which shows centroids for each variant of the studied variable (year, tillage and cropping system) represents the association between the spatial arrangement of the weed species in the field and the weight of the effects of the variables. Ordiplot A) shows how the year 2015–2016 had a significant influence on weed populations, mainly due to its rainfall distribution. Ordiplot B represents tillage systems and shows that the weed population was differently by NT. With respect to the cropping systems (ordiplot C), no appreciable differences were confirmed as both centroids, MON and ROT overlap.

## Discussion

### Influence of year, tillage and crop system on wheat yield parameters

Rainfall is a key climatic variable in Spain given the limited amount that falls in a great part of the territory and its high temporal and spatial variability. Spain exceeds 20% of interannual rainfall variability. Under Mediterranean arid and semi-arid conditions, crops are especially sensitive to periods of drought^45^. It is well demonstrated that the physiological stages of wheat are affected by climatic conditions, and rainfall and its seasonal distribution are especially determinant for wheat yield parameters^46^. Changes in rainfall patterns in rainfed cereals will affect weed biology, the weed community, and will influence the crop-weed interactions and crop growth patterns^47^.

Our results showed that wheat yield varied significantly according to the years of study. Consistent with the findings of other authors^48^ we have seen that the last year with maximum drought, ECE of drought, during wheat booting, anthesis and grain filling stages produced a negligible crop yield. In preceding studies, we have observed that high rainfall and mean temperatures in April, coinciding with wheat booting period, and were crucial for cereal growth^49^. There were no differences in crop biomass production between the three years of study, although in 2015–2016, the low rainfall in QR1 affected the weed germination, the crop biomass recovered thanks to following rainfall. Then, according to Shimshi et al.^50^ and previous experiences of our group^49^, moisture stress during tillering stage, corresponding in our study to QR2, influences wheat yield. Throughout our experiment, the efficiency of assayed systems, measured by means of the harvest index (HI), took normal ranges for wheat between 0.35 and 0.50^51^. Our data have also reflected the significant influence of year on HI, being the least efficient crop in 2016–2017, because of the strong influence of drought this year, considered an ECE of drought, which penalized the grain formation and filling. This result was in line with the wheat yield data variation observed during the years of study.

In accordance with Calzarano et al.^48^ and Alarcón et al.^52^, our data did not show significant effects between tillage systems on grain yield and straw biomass. The results of our study have suggested that final production did not vary, even with an ECE year of drought, probably because of environmental conditions were not advantageous to make a difference during the wheat growth cycle and the different tillage systems were unable to display their ability to mitigate the impact of the weather variations. On the other hand, Šíp et al.^53^ observed that low rainfall led to low yield but higher with NT than other systems, a situation that did not occur in our study, even under drought conditions. This inconsistency in the results was due to difference in growing management conditions due to the erratic rainfall distribution that induced variability in such results. In our short-term experiment, the tillage systems may have shown a different consistency than studies carried out in the long-term at the same location. If climatic conditions worsen in the short term, the effect of tillage systems on production is unsettled. However, we have seen that HI was significantly higher in MT compared to CT, which coincides with De Vita et al.^14^. In our experiment, the highest grain yield and the lowest straw biomass were recorded in MT meaning that this system seems to assure superior wheat grain formation and a quick mineralization of residues. Also, a significant reduction was observed in both the grain yield of wheat and its biomass in monoculture compared to rotation, which coincides with findings from other authors^54^. Our findings endorse the view of rotation crops a key tool within conservation agriculture techniques providing a well land management under semi-arid areas.

### Influence of year, tillage and crop systems on weed parameters

In general terms, the climatic conditions in the different years of this study had a significant influence on weed parameters (density, dry biomass, diversity). In 2015–2016, the very low rainfall in QR1, coinciding with the germination period of crop and associated weeds, reduced significantly the number of weeds per square meter, as well as species diversity. This can be explained by authors such as Schulte et al.^55^, since the germination and emergence of weeds is generally synchronized to start at the beginning of the humid period. Between QR2 and QR3 of 2015–2016, the total rainfall was equal to or higher than the historical average but with a different rainfall pattern, which allowed a development of weed biomass similar to 2014–2015. The year 2016–2017 had the highest weed parameter values (density, biomass, and diversity) coinciding with the lowest rainfall in QR2 and QR3 and the lowest production of wheat, which suggests a strong competition for water resources between crops and weeds that was clearly won by the weed community. Similar results were obtained by Calado et al.^56^ who indicated that the decrease in the relative yield was related to the increase in competition by weeds for limited resources such as water. In Mediterranean environments, Siddique et al.^57^ highlighted the role of evaporation from soil surface and the plant transpiration on the soil water content. It has been demonstrated that soil water dynamic was highly correlated with grain yield^58–60^. And Ryan et al.^33^ also suggested that weed assembly was influenced by soil hydrologic properties. López-Bellido et al.^61–63^ and previous findings in our long-term experiment^49^ recorded the influence of rainfall on soil water content during the wheat growth. And attributed higher yields to increased water conservation or efficient utilization by the crop with the NT-rotation systems.

The results reported here confirmed that NT significantly increased all weed parameters studied, (weed density, biomass, and diversity) compared to CT and MT. The NT system left the crop residues on top layer of soil preserved the water of rainfall and likely increased the soil water storage. This could have increased the water use efficiency for weeds in NT system. These results were consistent with foregoing experiments^23^ and López-Bellido et al.^62^, who observed higher soil water content in NT than in CT, although Cantero-Martínez et al.^26^ did not achieved an advantage of conservation system in terms of water efficient utilization by the crop in drylands. However, there is not a general agreement, and large differences have been reported by different authors probably due to different experimental conditions. In this context, Bilalis et al.^64^ recorded less weed species in NT than CT. According to the working conditions of Feledyn-Szewczyk et al.^65^ weed density was significantly higher in MT and Alarcón et al.^52^ found no differences between different systems. We noted that the ECE conditions of drought and erratic rainfall pattern in 2016–2017 favoured a general high weed density, which was especially higher in NT plots. Similarly, the weed biomass in NT in 2015–2016, the year with the lowest autumn rainfall, was higher than other tillage systems. These facts underline the NT as the system with the major water soil storage that can buffer stressful environmental conditions (as drought) and favour the presence of weeds.

In our study, the weed community in terms of biomass were significantly higher in wheat rotation than wheat monoculture systems, although weed density did not vary. The results contrast with those obtained by Woźniak^54^ with an increase of weeds in wheat monoculture compared to rotation. In our experiment, a perennial weed, *Cardaria*, significantly increased its biomass at rotation system probably favoured by the previous fallow that retained the soil water storage to facilitate the weed growth. However, despite the drought conditions, the greater weed biomass did not reduce yield in rotation system. These results highlight the role of the previous crop on the weed emergence and support the idea of using rotation as a tool to keep yield crop and soften the effects of drought conditions. Furthermore, results by Pala et al.^66^ have pointed the usefulness of rotation in a crop system can be determined by the rotation crop’s water use efficiency and its ability to adapt to drought stress.

Over the study, values reported for Shannon’s index were lower than 2 and reflected less diversity in accordance with values reported in other studies in Mediterranean regions^67^. The highest values of this index were found in NT systems, which was significantly different from MT (the lowest value), on the other hand, CT did not differ significantly from either. On the contrary, Feledyn-Szewczyk et al.^65^ found the MT tillage system to have the highest diversity index with humid soil conditions due to great annual precipitation. In other studies^68^, the tillage system in organic farming had no impact on the Shannon index. In addition, cropping system had no impact on weed diversity, contrary to the findings of Sarani et al.^69^. The Pielou’s index has been found to suggest that intermediate values (0.4–0.8) mean that there are no dominant species^67^. All the Pielou index values obtained in our experiment were above 0.7, meaning that there were no dominant species in the fields. Year and the crop system had no influence on this index; however, CT had the highest weed species evenness, being significantly different to MT and NT tillage systems. These results agree with Pardo et al.^70^ who considered that the action of ploughing—moving the soil—in CT favoured uniformity among the species present in the seed bank, thus avoiding the dominance of a single species. Similarly, Sans et al.^68^ observed a reduction in the values of evenness in plots with reduced tillage. The variety of findings in the literature are due to weed diversity short-term studies have been subjected to changes by weather conditions and agronomic practices. Though these contrasting results, we highlighted the relevance of these short-term studies in the site-specific weed management.

Focusing on the principal weed species in our study, the factor with the highest incidence on weed community dynamics had been the environmental conditions, followed by the tillage system and finally the crop rotation. The rainfall pattern affected the emergence of plants and was determinant in the appearance of autumn–winter weed species. According to the results of a study by Calado et al.^56^, the low rainfall in the autumn winter months (QR1) of 2015–2016, in our case 44 mm, caused a reduction of 70% in weed density and 50% in weed diversity compared with the other two years of study, as we can see in our study. Less weed species emerged in the field and the competition for water resources was diminished. Bearing this in mind we observed that the densities of *Capsella, Hypecoum, Papaver* and *Lolium* were clearly diminished the year 2015–2016, a year with the lowest rainfall in QR1. So, this low rainfall period affected the germination of autumn–winter species (Santín-Montanyá et al., 2013). On the contrary, *Cardaria* density increased probably because of the reserves accumulated in the rhizomes allow it to survive better, despite minimal autumn rainfall in 2015–2016, and so the plant is able to survive. Moreover, the accumulated annual rainfall in 2015–2016 facilitated the emergence of this perennial specie and its biomass increased likely due to the soil water storage during the fallow period.

Regarding the tillage systems, the NT system favoured *Anacyclus, Capsella; Galium; Hypecoum; Lamium* and *Papaver* as expected because they all have small-medium seeds that germinate on the surface^71^. *Cardaria*, a perennial weed with rhizomes, was favoured by CT, which contrasts with the findings obtained by Sans et al.^68^. The soil tillage could break the rhizomes of this species and facilitate its dispersion^72^. We have also found two species, *Anacyclus* and *Fumaria,* were more abundant in monoculture than rotation systems, where these species were well controlled because the fallow management previously to wheat favoured the emergence of these weeds during the fallow period. *Cardaria* was more prolific in rotation systems than in monoculture. This perennial species has deep, pivoting roots which means that as it outlives the rotation crop the roots are established and there is little competition with the newly seeded cereal crop for water. Coincident with Ruisi et al.^21^ findings, the weed population in a cereal field can be affected by the crop system because of weed control measures from previous years, which depend on the type of rotation crop. We also observed that *Anacyclus* density was higher in NT and monoculture, and NT and high autumn rainfall favoured *Lamium.* These results confirm that annual species dynamics predominant in a field can change with the agronomic practice selected under dryland areas with low/erratic rainfall. Regarding perennial species as *Cardaria* density was affected by the year, tillage and crop systems interactions in a different way than the rest of annual weed species selected. So, this weed species, with pivoting roots that take up the deep water and with rhizomes able to survive severe drought periods, would thrives at CT and rotation systems in spite of low autumn rainfall.

We have found that seasonal rainfall and land uses had a great influence on wheat yield and the dynamic of weed populations in the short-term. GSR was the more influential variable on wheat production and weed community. As were expected, the last year of our study, defined as ECE, had the lowest wheat production and the highest weed presence. We have also seen that year had a great impact on the weed community: high autumn rainfall (QR1) facilitated the weed germination and weed diversity increased, while low rainfall during QR2 and QR3 reduced the weed parameters and the crop suffered also by drought. The influence of management variables, tillage and crop systems, were significantly important too. In this regard, NT- rotation combination techniques maintained the wheat yield despite of the increase weed community under ECE of drought.

## Conclusions

We conclude that changes in climatic conditions amplified the uncertainties in wheat yield variability as well as variations in weed density and diversity. The regions currently classified as semi-arid, climate change is forecasted to lead to more erratic rainfall patterns, and ECEs will become frequent in the long-term. In this research, changes were observed in the short-term, but persistent low/erratic rainfall may be a driver to adopt the conservation agriculture technologies, particularly those years with high drought (ECE). In this context, specific-site studies can be very useful to farmers when they need to select and/or combine conservation agriculture technologies during several campaigns. Our short-term study held that residues left on soil surface (in NT) and the fallow period (in crop rotation system) increased the weed pressure but did not diminished the wheat yield compared to conventional systems even with an ECE for drought.

Then, knowledge of crop and weed behaviour under different environmental and management systems is key to maintaining sustainable rainfed agriculture under changing climate conditions. We consider that some functional attributes of weeds (such as the roots, size of seeds etc.…) play an important role in their ability to thrive and further studies would be necessary to optimise weed management techniques accordingly.
